# DESM: portal for microbial knowledge exploration systems

**DOI:** 10.1093/nar/gkv1147

**Published:** 2015-11-05

**Authors:** Adil Salhi, Magbubah Essack, Aleksandar Radovanovic, Benoit Marchand, Salim Bougouffa, Andre Antunes, Marta Filipa Simoes, Feras F. Lafi, Olaa A. Motwalli, Ameerah Bokhari, Tariq Malas, Soha Al Amoudi, Ghofran Othum, Intikhab Allam, Katsuhiko Mineta, Xin Gao, Robert Hoehndorf, John A. C. Archer, Takashi Gojobori, Vladimir B. Bajic

**Affiliations:** 1 King Abdullah University of Science and Technology (KAUST), Computational Bioscience Research Center (CBRC), Thuwal 23955-6900, Kingdom of Saudi Arabia; 2 New York University, Abu Dhabi, UAE; 3 King Abdullah University of Science and Technology (KAUST), Center for Desert Agriculture (CDA), Thuwal 23955-6900, Kingdom of Saudi Arabia; 4 King Abdullah University of Science and Technology (KAUST), Biological and Environmental Sciences and Engineering Division (BESE), Thuwal 23955-6900, Kingdom of Saudi Arabia; 5 King Abdullah University of Science and Technology (KAUST), Computer, Electrical and Mathematical Sciences and Engineering Division (CEMSE), Thuwal 23955-6900, Kingdom of Saudi Arabia

## Abstract

Microorganisms produce an enormous variety of chemical compounds. It is of general interest for microbiology and biotechnology researchers to have means to explore information about molecular and genetic basis of functioning of different microorganisms and their ability for bioproduction. To enable such exploration, we compiled 45 topic-specific knowledgebases (KBs) accessible through DESM portal (www.cbrc.kaust.edu.sa/desm). The KBs contain information derived through text-mining of PubMed information and complemented by information data-mined from various other resources (e.g. ChEBI, Entrez Gene, GO, KOBAS, KEGG, UniPathways, BioGrid). All PubMed records were indexed using 4 538 278 concepts from 29 dictionaries, with 1 638 986 records utilized in KBs. Concepts used are normalized whenever possible. Most of the KBs focus on a particular type of microbial activity, such as production of biocatalysts or nutraceuticals. Others are focused on specific categories of microorganisms, e.g. streptomyces or cyanobacteria. KBs are all structured in a uniform manner and have a standardized user interface. Information exploration is enabled through various searches. Users can explore statistically most significant concepts or pairs of concepts, generate hypotheses, create interactive networks of associated concepts and export results. We believe DESM will be a useful complement to the existing resources to benefit microbiology and biotechnology research.

## INTRODUCTION

An overwhelming amount of literature is associated with the microorganism research area, as they are of special interest to industry and bring about cycling of nutrients and compounds essential for the survival of all organisms (1). These microorganisms are found to inhabit diverse environments, some more extreme than others, and thus have adapted or developed mechanisms of resistance that allow them to find energy, digest food and reproduce (2–4). In this process, a variety of chemical compounds are produced. These diverse microbial activity mechanisms are being used in the production of food, agriculture, petrochemical and biotechnology industries, medicine and warfare. Some examples include the use of: (i) microbes to produce dairy, meat, fish, vegetables, legumes, cereals, beverages and vinegar (5), (ii) microbes to alter plant DNA conferring resistance to insects and viruses (6), (iii) plants root bacteria to convert nitrogen from the air into a form that the plant can use, like fertilizer, (iv) decomposing microbes in wastewater treatment plants, composting facilities and landfills (7), or production of antibiotics (8).

For bacteria, there are currently approximately 12 000 draft and complete genomes that are annotated. Additionally, some microorganisms are adjusted through directed evolution or engineered for industrial production of important chemicals, which makes them the so-called microbial cell factories. Thus, it is of general interest for microbiology and biotechnology researchers to have means to explore information about molecular and genetic basis of functioning of different microorganisms, as well as their ability for bioproduction, seen from the viewpoint of interconnections/associations of different concepts such as chemical compounds, existing functional annotation, genes/proteins and taxonomy. Associations between concepts can be used to generate networks of concept links that can be conveniently visualized, which can help in understanding: (i) potential influences these entities may have on each other, (ii) functioning of the microorganism as a whole and/or (iii) its capacity for specific bioproduction.

To enable such information exploration, we compiled 45 topic-specific knowledgebases (KBs) focused on different aspects of microbial activities or microbial groups. These KBs are accessible through DESM portal (www.cbrc.kaust.edu.sa/desm), which is a platform for discovery, analysis and exploration of information from these topic-specific microbial-focused KBs. The KBs are compiled using integration of text-mining and data-mining and they address the task of association discovery with respect to the specialized but broad spectrum of topics related to microorganisms. In DESM, for the compilation of the background information, the text-mining part utilized all PubMed records indexed with 4 538 278 concepts from 29 dictionaries. The 45 topic-specific KBs in DESM used information from 1 638 986 of these indexed PubMed records. Information exploration is enabled through searches of the text-mined information and its integration with data-mined information from external data sources. A more detailed description of DESM follows.

## DATABASE COMPILATION

### Overview of DESM design

DESM data are hosted on a PostgreSql server and queried through an interactive web interface implemented using PHP and JQuery. The portal provides access to different KBs that run under the control of DES v2.0, an in house knowledge exploration platform developed from the previous version of Dragon Exploration System (DES). Previous versions of DES have been used for the compilation of a number of databases and KBs (9–15), as well as for several patent pending discoveries based on the mined information. DES v2.0 is a web-based, three-tier application (Supplementary Figure S1) consisting of data, logic and presentation tiers. Data are stored and processed by the data tier that is implemented in open source, PostgreSQL object-relational database system. DES uses highly structured data divided into number of databases and multiple schemas to group similar tables, indexes and functions. The logic tier utilizes open source, Apache-based web and application server. In addition to server scripts, the application server hosts a number of indexing and background data processing utilities. The presentation tier is AJAX-based (asynchronous JavaScript and XML), which allows for efficiency and provides the user with a features rich, easy to use interface.

### Construction of individual KBs

The DES system relies on two main components: controlled vocabularies (dictionaries), which comprise concepts from specific fields, and PubMed records. The text of all titles and abstracts of scientific publications referenced in PubMed is matched against all dictionaries locally to provide a global index used in DESM to link each concept to its occurrences within the PubMed records. This indexing is done at the character level to enable concept highlighting within a sentence, as well as at the document (title and abstract) level to enable concept occurrence and co-occurrence counts. To create a KB, the KB index is created from this global index by restricting it to PubMed identifiers retrieved as a response to the PubMed query that defines the topic of the KB (Supplementary Figure S2). From this KB index, a number of relations are derived at the KB creation time such as enriched concepts, enriched associations between these concepts and potential hypotheses. The enrichment refers to over-representation of the concepts or pairs of concepts in the KB as compared to the whole PubMed. This enrichment is characterized by the default false discovery rate (FDR) of 0.05.

Most of the compilation of a KB is automated. The manual procedures involve: (i) generation of the query for retrieving the relevant PubMed IDs, (ii) selection of relevant dictionaries and (iii) possible additional cleaning of dictionaries by elimination of promiscuous terms.

### Populating KBs

Each KB is generated from titles and abstracts retrieved from PubMed records in response to a specific query, and complemented by data-mined information from a number of major resources from biology fields, such as ChEBI (16), Entrez Gene (17), GO (18), KOBAS (19), KEGG (20), Reactome (21), UniPathways (22), PANTHER (23) and BioGrid (24). Text-mining is performed using 20–29 different dictionaries (depending on KB) that include controlled vocabularies of relevance for the KB's specific topic. In DESM, some KBs focus on selected microbial activities, e.g. production of important compounds such as ethanol, butanol, acetone, nutraceuticals or biocatalysts, while others are focused on selected categories of microorganisms, e.g. lactobacillus, streptomyces, sulphur-reducing bacteria or cyanobacteria, which have found use in industrial applications.

## INFORMATION CONTAINED IN KBs

### Concepts and dictionaries

The information contained in a KB is seen in DESM through the concepts identified in the analyzed topic-specific set of documents, as well as through the potential associations between these concepts. These concepts are terms collated into category-specific dictionaries. For example, there are dictionaries for ‘Industrially Important Enzymes’, ‘Chemical Entities of biological interest’, ‘Antibiotics’, etc. (the list of the dictionaries we compiled and used in DESM is given in Table 1). One concept can appear in various versions in a free text. Thus, to keep non-redundant information, concepts are normalized (i.e. only one index internally in DESM would represent the concept that may appear in various versions of names, synonyms and symbols that would all describe the same entity). Concepts in all dictionaries (except for ‘Pathways’, ‘Human Anatomy’ and ‘Geographical Names’) are normalized (Table 1).

**Table 1.** Dictionaries used in DES v2.0

Dictionary_name	Dictionary_category	Source	New/updated	Normalized
Archaea (NCBI Taxonomy)	Taxonomy	Entrez Taxonomy	new	yes
Bacteria (NCBI Taxonomy)	Taxonomy	Entrez Taxonomy	new	yes
Fungi (NCBI Taxonomy)	Taxonomy	Entrez Taxonomy	new	yes
Marine Snails (NCBI Taxonomy)	Taxonomy	Manually compiled and curated	updated	yes
Porifera taxons	Taxonomy	Manually compiled and curated	updated	yes
Source Microbes for Antibiotics	Taxonomy	Manually compiled and curated	new	yes
Viroids (NCBI Taxonomy)	Taxonomy	Entrez Taxonomy	new	yes
Viruses (NCBI Taxonomy)	Taxonomy	Entrez Taxonomy	new	yes
Archaea Genes (EntrezGene)	Genes/proteins/transcripts	Entrez Gene	new	yes
Bacteria Genes (EntrezGene)	Genes/proteins/transcripts	Entrez Gene	new	yes
Fungi Genes (EntrezGene)	Genes/proteins/transcripts	Entrez Gene	new	yes
Viroid Genes (EntrezGene)	Genes/proteins/transcripts	Entrez Gene	new	yes
Viruses Genes (EntrezGene)	Genes/proteins/transcripts	Entrez Gene	new	yes
Biological Process (GO)	Functional annotation	GO	updated	yes
Cellular Component (GO)	Functional annotation	GO	updated	yes
Disease Ontology (DO)	Functional annotation	DO	updated	yes
Molecular Function (GO)	Functional annotation	GO	updated	yes
Pathways	Functional annotation	KEGG, Reactome, UniPathway, Panther	updated	no
Antibiotics	Chemicals/Compounds	Manually compiled and curated	updated	yes
Chemical Entities of Biological Interest (ChEBI)	Chemicals/Compounds	ChEBI	new	yes
Conopeptides	Chemicals/Compounds	Manually compiled and curated	updated	yes
Drugs (DrugBank)	Chemicals/Compounds	DrugBank	new	yes
Enzymes (Intenz)	Chemicals/Compounds	Intenz	new	yes
Industrially Important Enzymes (EC)	Chemicals/Compounds	Manually compiled and curated	new	yes
Metabolytes (Metabolights)	Chemicals/Compounds	Metabolights	new	yes
Sponge Compounds	Chemicals/Compounds	Manually compiled and curated	updated	yes
Toxins (T3DB)	Chemicals/Compounds	T3DB	new	yes
Geographic Names	General	Manually compiled	updated	no
Human Anatomy	General	Manually compiled	updated	no

The sources used to compile dictionaries are listed in Table 1. There are a number of databases that provide nomenclatures for entities in various fields, e.g. Entrez Gene provides a taxonomy-based nomenclature for genes, which includes: the gene official name and alternative names, official symbol, aliases, etc. For one gene, these are provided related to an Entrez Gene unique identifier. Some other concepts are derived from the nomenclatures that are in the form of ontologies, such as ‘Gene Ontology (GO)’ and ‘Disease Ontology (DO)’. The third group of dictionaries is derived from the taxonomy information contained in Entrez Taxonomy database, such as for ‘Achaea (NCBI Taxonomy)’, ‘Bacteria (NCBI Taxonomy)’, ‘Fungi (NCBI Taxonomy)’, ‘Viruses (NCBI Taxonomy)’, and ‘Viroids (NCBI Taxonomy)’. Finally, other dictionaries such as ‘Antibiotics’, ‘Conopeptides’, ‘Sponge Compounds’, ‘Porifera Taxons’, ‘Source Microbes for Antibiotics’, ‘Marine Snails’, ‘Geographic Names’ and ‘Human Anatomy’ (Table 1) are derived manually from the relevant literature and public resources, and curated, except the last two. All dictionaries are further cleaned from the ‘common’ English terms and after that manually cleaned by eliminating promiscuous terms based on the frequency of their appearance, so as to reduce the ‘noise’ in the DESM reports.

Furthermore, in DESM, when dictionaries are compiled from various sources we integrated them into a unified format, in our case a relational database schema. This schema keeps track of the imported concepts, including their constituent terms, and the dictionaries they are assigned to. This schema also allows normalization by keeping track of term source database identifiers.

When a single term is shared by multiple concepts within the same dictionary it becomes ambiguous. Therefore, such terms are excluded from that dictionary. In this case, the corresponding concept can still be text-mined through other versions of its name. However, terms or whole concepts can appear in several dictionaries, e.g. proteins and enzymes, chemicals and drugs, genes and disease, etc. In such cases, an index record is created for the same term for each dictionary it belongs to.

When PubMed document title and abstract are presented, the terms are highlighted in different colors for easier recognition of concepts belonging to different dictionaries.

When concepts are presented in a tabular format, only those that are statistically enriched in the KB are listed, so as to increase chances to keep those most relevant to the KB. This is achieved by determining the *P*-value for concept enrichment in the KB as opposed to the whole PubMed. The *P*-values are calculated based on the hypergeometric test for enrichment. This *P*-value is corrected for multiplicity testing based on the Benjamini–Hochberg method (25). Note that this *P*-value is also known as FDR. We used a default FDR of 0.05. The concepts are by default ranked based on FDR. In all cases in DESM the *P*-values are determined as described above.

### Associations of concepts

An association between the concepts A and B is any formal connection/link between the concepts. In order for an association to be useful, it should be meaningful. This means the link between the concepts A and B should make sense in a specific context. Thus, to increase the chances for this, in DESM we rely on the assumption that two concepts A and B have more chances to be mutually dependent/associated/linked if they co-occur (in the same document) within the context of the KB more than would be expected by chance. To provide for this, in DESM, only the co-occurrences of the statistically enriched concepts in the same documents are used to compute two measures of association: Point-wise Mutual Information (PMI) (26) and FDR. These measures of the strength of association are used in DESM to rank the co-occurring concepts. We used a default FDR of 0.05 to list the potential associations.

### Hypotheses

Consider concepts A, B and C. If concept A and B are associated, and concepts B and C are associated, according to the Swanson linking technique (27) it can be hypothesized that concepts A and C are also associated by transition, even if this information is not directly reported. For example, if an association between disease A and protein B is reported in one document (e.g. protein B is highly expressed in disease A), and another document reports an association between protein B and drug C (say drug C inhibits expression of protein B), then, it can be hypothesized from these two pieces of disjoint data that drug C might have an effect on disease A. Swanson linking is performed in DESM and indirect links representing potential hypotheses are reported.

## IMPROVEMENTS INCORPORATED IN DES V2.0

DES v2.0 is a completely new redevelopment and is significantly advanced compared to the previous versions of DES. These extensions include:

- A significant expansion of the controlled vocabularies: Some new dictionaries, not present in the previous versions, were added, and some of the existing dictionaries were updated (Table 1). This led to a 6-fold increase in the number of terms used for document indexing (from less than one million to over six million terms).
- Various optimizations: The significant size of the resulting index affects backend processes such as indexing time, KB creation/rebuilding time, as well as KB queries (frontend responsiveness). A number of optimizations were implemented to speed up KB queries. Backend processes were also significantly optimized to enable easier periodic data cleaning, re-indexing and KB rebuilding within reasonable time frames. This also included a hardware upgrade of the database host.
- Concept normalization: Normalization enables the capturing of term variations of the same concept in text, and assigning these alternatives to the same standard identifier that can be recognized by external data sources (e.g. Entrez gene identifier). Previous versions of DES lacked this feature, but newly compiled and updated dictionaries within DES v2.0 contain normalized concepts in almost all cases. Any further extension of DES v2.0 will also implement normalization of concepts.
- Concept and association enrichments: Term frequencies were used in previous DES versions to rank terms and their co-occurrences. This led to common terms (e.g. ‘protein’) and their corresponding associations to be highly ranked, even if they have little relevance to the KB or they represented too general concepts to convey useful information in the context of a specific KB. In DES v2.0, concepts and associations are ranked based on how much they are ‘over-represented’ within the specific KB. Concept normalization has helped to more accurately determine the enrichments.
- Inclusion of external information: Normalization enables linking text-mined data to external data sources. Within DESM in particular, KOBAS pathways were enriched based on gene/protein mentions within the text. So currently, there is a wealth of information that is incorporated within DES v2.0, such as gene–gene interactions (BioGrid), gene-pathway associations (KOBAS), GO ontology enrichments based on gene/protein mentions (GO annotations).
- DES v2.0 web interface and the network viewer also underwent a number of changes, which were mainly based on feedback from users of the system. The aim was to make these simple, intuitive and easy to use. Network representation is based on Cytoscape (28) and other graphical representations of concepts and associations are based on Krona (29).
- In the original version, the PubMed documents were retrieved and indexed on the fly. In DES v2.0 the whole local installation of PubMed is indexed in advance, and only the PubMed identifiers of the topic-specific PubMed records are obtained by querying PubMed directly.

## UTILITIES

DESM provides users with a number of tools to explore, filter and visualize enriched concepts and their associations. The instructions are provided on the KBs help pages. Users have possibility to explore statistically most significantly enriched concepts from numerous used dictionaries. It is possible to find associations of a particular concept from all or specific dictionaries, to explore pairs of concepts, as well as to generate hypotheses. The networks of associated concepts can be incrementally built and interactively adjusted. In all scenarios, the concepts or pairs of concepts are ranked based on the *P*-values corrected for multiplicity testing, point-wise mutual information or number of PubMed documents where concepts are found. Concepts from most of the dictionaries are normalized. Some concepts such as pathways are not normalized due to the disparities of pathway contents when pathways appear in different repositories. The help instructions are provided. Users have possibility to export many types of information of interest. As an example, if KB for production of biocatalysts is considered, one can find information linking genes/proteins from bacteria and archaea, bacterial and archaea species, different pathways, metabolites, enzymes, toxins, etc. to help exploring underlying mechanisms of biocatalysts production across various microorganisms.

In order to access any of the KBs users have to click on the ‘Open Knowledgebases’ tab from the main menu on the top of the DESM homepage. Then any of the KBs can be selected from the left side table and opened by clicking on the ‘Open’ button at the right top of the page. The content of the KB can be explored through the ‘Concepts’, ‘Associated Concepts’, ‘Hypotheses Explorer’ and ‘KOBAS Pathways’ links on the left side menu. Here we briefly describe each of them.

### Concepts

Concepts can be ranked by *P*-value, frequency of appearance in KB (KB frequency), frequency of appearance in the whole PubMed (PubMed frequency) or alphabetic order. As the concepts are normalized, a number of terms may represent the same entity. In the literature view, a concept is expanded to display synonyms, and its occurrences are highlighted within the text according to a dictionary-based color-scheme. Concepts can be filtered using the search functionality, by dictionary or by restricting their *P*-value, or raw counts. Concepts also have a right click menu to bring up their associated concepts in tabular format, as a pie chart or as a Cytoscape (28) network.

### Associated concepts

Concept pairs can be ordered by *P*-value, PMI or co-occurrence counts. Similarly, associations can be filtered by searching on one or both contributing concepts by, (i) dictionary, (ii) restricting the *P*-value, PMI or (iii) the term co-occurrence frequency.

### Hypotheses explorer

Ranking of the most promising hypotheses as explained above.

### KOBAS pathway (KEGG Orthology Based Annotation System)

Even though a number of pathway sources are used to compile the pathway dictionary in DESM, only a small proportion gets matched to the text. In particular, long name pathways have a higher probability to have text variations and consequently be missed by the parser. In DESM, taxonomy specific pathway enrichment is also provided through the use of external gene-to-pathway annotations.

KOBAS (**K**EGG **O**rthology **B**ased **A**nnotation **S**ystem, http://kobas.cbi.pku.edu.cn) provides such annotations which integrate a number of pathway databases, namely: KEGG PATHWAY, PID (30), BioCarta (31), Reactome, BioCyc (32) and PANTHER (23). Over-represented pathways for a particular taxonomic category are identified by first extracting the genes within the knowledge-base belonging to the taxonomy, and using that as a sample input against the corresponding KOBAS annotation as background for calculating the enrichment *P*-values. Separately from these, each concept can be explored also through the graphical interactive network view. This is accessible by right click on the term of interest and choosing the ‘Network’ option.

### Network view

Users can be interested in various scenarios involving a number of concepts from various dictionaries, where these scenarios are mostly set out with exploratory tasks that consequently develop into targeted investigations or curation tasks. Sifting through term pairs in tabular format is not always the best option and the network viewer is more suited for this kind of general-purpose exploration. Using the network view, the user can incrementally build a network of concepts and their associations, by choosing one or more dictionaries at each step, and trimming out irrelevant links as they progress. The nodes in the network represent concepts and they are color-coded and assigned different shapes to allow for an easier visual distinguishing of various types of concepts. The ‘Help’ page explains the use of the network view.

## EXAMPLES OF POTENTIAL USE

### Identification of candidate antitubercular drugs via drug repositioning

To demonstrate how DESM can be used to possibly identify drugs suitable for repositioning, we consider identifying a candidate drug to treat tuberculosis. Studies show that when oxygen and nutrients are depleted, the tricarboxylic acid cycle (TCA) is down-regulated and the alternate glyoxylate cycle sets in to produce energy (33). Moreover, it has been demonstrated that during down-regulation of TCA cycle, inhibition of the glyoxylate cycle enzyme, isocitrate lyase, is fatal to *Mycobacterium tuberculosis* (34). *Mycobacterium tuberculosis* is the infectious agent for tuberculosis disease, the greatest killer worldwide only second to HIV/AIDS (35). Thus, scientific research has been focused on isocitrate lyase as potential drug target for the identification of new antitubercular drugs. However, *Mycobacterium tuberculosis*/isocitrate lyase-related research is sluggish owing to it requiring biosafety level three facilities and *Mycobacterium tuberculosis* itself being slow growing. To support *Mycobacterium tuberculosis*/isocitrate lyase-related research, DESM can be used to possibly identify candidate antitubercular drugs via drug repositioning.

Here, we take into account that the role of isocitrate lyase in bacterial and fungal pathogenesis has been reported (36). Some example include: (i) pathogenesis of fungus *Leptosphaeria maculans* upon infection of canola (*Brassica napus*) (37), (ii) pathogenesis of fungus *Magnaporthe grisea* upon infection of rice blast (38) and (iii) pathogenesis of fungus *Candida albicans* upon infection of the human host (39). Thus, an anti-fungal drug that targets isocitrate lyase directly or indirectly may be a candidate drug that can be repositioned for tuberculosis treatment.

*C. albicans* is a common pathogen while *Saccharomyces cerevisiae* is rarely found in human hosts. Even so, both fungi are readily phagocytosed by macrophages. Macrophages efficiently kill *S. cerevisiae*, while *C. albicans* cells grow in a filamentous morphology thereby killing macrophages in the process. Nonetheless, *S. cerevisiae* has been used as a model organisms to study fungal primary response to phagocytosis; it was observed that enzymes of the glyoxylate cycle where highly induced including key enzymes, isocitrate lyase and malate synthase (40). Based on these observations for *S. cerevisiae*, it is interesting to analyze the glyoxylate pathway in *C. albicans* when this organism is inside the macrophage. *C. albicans* homologs of isocitrate lyase were induced upon phagocytosis (40). Thus, for the below drug repositioning demonstration we use *S. cerevisiae* because it has been used as a model organism to study fungal phagocytosis, shown to induce isocytrate lyase in this process and will likely not provide a patentable drug but instead provide a mere plausible demonstration of how DESM can be used to derive candidate drug that could be repositioned.

#### Drug repositioning demonstration

**Figure 1.**
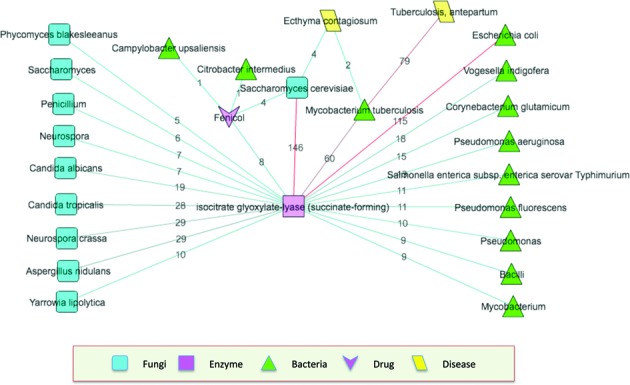
A step-wise illustration of how DESM can be used to short-list candidate antitubercular drugs via drug repositioning. This search focused toward identifying drugs that target isocitrate lyase for the treatment of tuberculosis, where 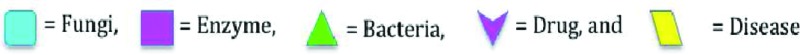. The numbers associated with edges indicate the number of PubMed documents where the concepts linked by the edge co-occur.

The ‘DESM_Isocitrate_Glyoxylate_Lyase’ Knowledgebase is used for this demonstration. Highlight the ‘DESM_Isocitrate_Glyoxylate_Lyase’ Knowledgebase, then click ‘Open’ in the right side pane. Select the term ‘isocitrate glyoxylate-lyase (succinate-forming)’, then right click to generate a ‘network’. Select ‘isocitrate glyoxylate-lyase (succinate-forming)’, and expand its association with the ‘Fungi’ and ‘Bacteria’ dictionaries. Then, select ‘Saccharomyces cerevisiae’ (one of the fungi retrieved), and expand its association with the ‘Enzyme’, ‘Disease’ and ‘Drug’ dictionaries. Similarly, select ‘*Mycobacterium tuberculosis*’, and expand its association with the ‘Enzyme’, ‘Disease’ and ‘Drug’ dictionaries. For the ‘Fenicol’ drug, expand its association with the ‘Enzyme’, ‘Disease’ and ‘Bacteria’ dictionaries. Select all enzymes, drugs and diseases except ‘Tuberculosis, antepartum’, ‘Ecthyma contagiosum’, ‘Fenicol’ and ‘isocitrate glyoxylate-lyase (succinate-forming)’, right click to remove selected terms (Figure 1). Figure 1 demonstrates that ‘Fenicol’ is associated with ‘Saccharomyces cerevisiae’ and ‘isocitrate glyoxylate-lyase (succinate-forming)’ and is not linked to ‘*Mycobacterium tuberculosis*’, even though ‘*Mycobacterium tuberculosis*’ is linked to ‘isocitrate glyoxylate-lyase (succinate-forming)’. This finding suggests that ‘Fenicol’ is a plausible candidate drug that can be considered for treatment of *Mycobacterium tuberculosis*-associated diseases, ‘Tuberculosis, antepartum’ and ‘Ecthyma contagiosum’, if these associations cannot be found in a literature search as well. Figure 1 only shows the network based on the ‘Fenicol’ drug (for visual simplicity), however, several drugs are usually associated with each fungi and bacterium. This procedure can be applied to the other fungi and bacteria not tested, to generate a list of plausible candidate drug that can be considered for treatment of *Mycobacterium tuberculosis*-associated diseases. However, it must be noted that all drugs candidates short-listed in this manner must be further verified with a literature search.

### Archaea in the human body

Members of the domain Archaea have been chronically neglected when compared with Bacteria, but research in the field is currently witnessing a wave of new discoveries and renewed interest in their diversity, ecology and applications (41). The widespread use of molecular-based methodologies was vital to this shift. Most importantly, they showed that Archaea were much more diverse and ubiquitous than anticipated challenging the long-standing perception that they were restricted to extreme environments. Indeed, they populate and thrive in a variety of moderate environments, are involved in symbiotic relationships, and surprisingly have even been detected in several parts of our bodies (42–46). Some studies point to a possible link between their presence and certain medical conditions (47–49).

The network view tool in the ‘DESM_Human_Microbes’ KB is used to illustrate the relationship between archaea and human anatomy. This demonstration includes: (i) selecting an appropriate sub-group of parts of the human body, after multiple rows of expansion based on the human anatomy dictionary; (ii) expanding each human anatomy term using the Archaeal taxa dictionary, so as to highlight the co-occurrence of specific archaea within each location; (iii) expanding each obtained archaeal taxa using the Disease ontology dictionary to link the location in the human body, and presence of archaea to a specific disease.

**Figure 2.**
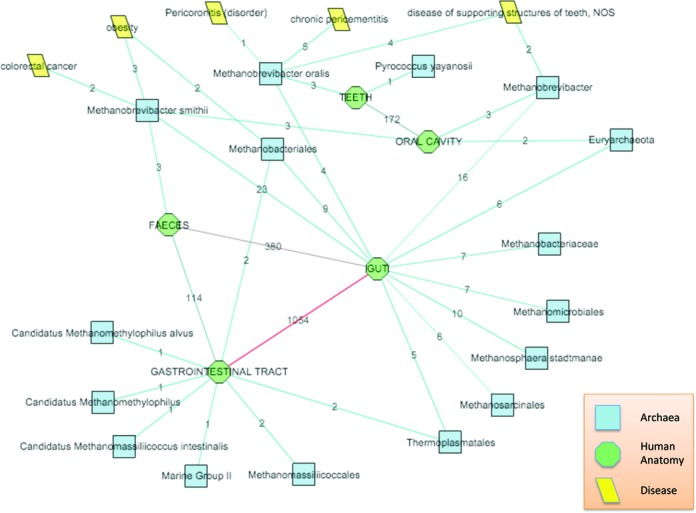
An illustration of how DESM can be used to quickly screen existing bibliography and confirm that most co-occurrences of Archaea and the human body are linked to the oral cavity, the gastro-intestinal tract and feces (or closely related terms), and that the vast majority of taxa belonged to the methanogens, where 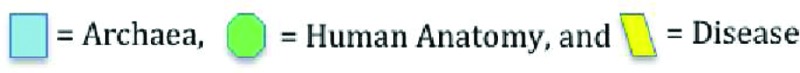. The numbers associated with edges indicate the number of PubMed documents where the concepts linked by the edge co-occur.

The network view generated using DESM (Figure 2) allowed us to quickly screen the existing bibliography and confirm that most co-occurrences of Archaea and the human body are linked to the oral cavity, the gastro-intestinal tract and feces (or closely related terms), and that the vast majority of taxa belonged to the methanogens. The highest number of co-occurrences at the species level consisted of *Methanobrevibacter smithii*, *Methanosphaera stadmanae* and *Methanobrevibacter oralis*. Furthermore, the species of the genus *Methanobrevibacter* were seemingly linked with several human diseases, which were directly related to their preferential location in the human anatomy (e.g. Methanobrevibacter oralis was linked to teeth, and to three different dental pathologies).

The prevalence of methanogens, as the most abundant archaea in human bodies, as well as the higher incidence of these three species is in good agreement with previous studies (47,48,50). *Methanobrevibacter smithii* is widely recognized as the most abundant archaea in our bodies, most importantly in our grastrointestinal tract (51), while *M. oralis* is the dominant archaeal species in the oral cavity (52,53). Also, the link between the aforementioned *Methanobrevibacter* species and these pathologies has been previously noted by other researchers and is at the center of several recent studies and ongoing debate on the exact role of archaea, which might be linked to syntrophy (49,50).

**Figure 3.**
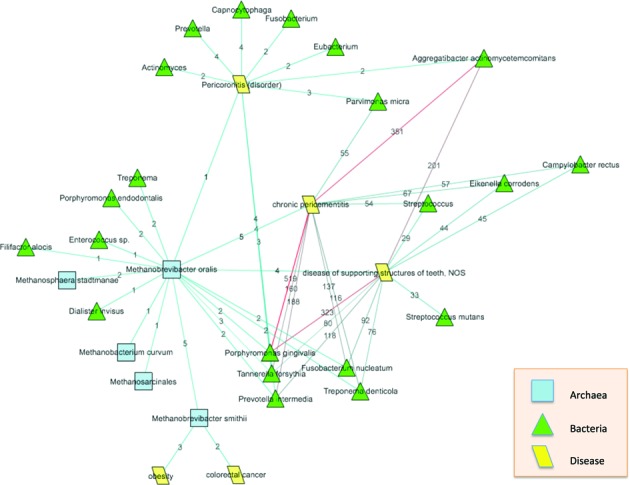
An illustration of how to generate a more topic-specific network, instance specifically on *Metanobrevibacter oralis* and associated diseases (chronic peridementitis, pericoronitis and disease of supporting structures of teeth), we quickly identified a network of associated microbial and archaeal taxa, where 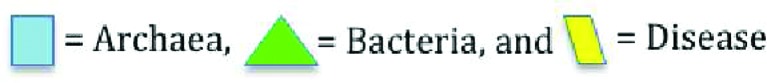. The numbers associated with edges indicate the number of PubMed documents where the concepts linked by the edge co-occur.

Focusing our attention specifically on *Metanobrevibacter oralis* and associated diseases (chronic peridementitis, pericoronitis and disease of supporting structures of teeth), we quickly identified a network of associated microbial and archaeal taxa (Figure 3). An interesting observation was the co-occurrence of specific bacterial species that were simultaneously linked to *M. oralis* and to one or more of the three dental pathologies. Particularly noteworthy within this community were *Porphyromonas gingivalis*, *Tannerella forsythia* and *Prevotella intermedia*, as they were linked to all three pathologies, and to *M. oralis*. Fittingly, a recent study discussed possible direct and indirect interactions between 10 bacterial species intimately associated with periodontitis, which included the three bacterial species listed above, and *M. oralis* (49).

The knowledge of the microbial network associated with specific diseases is vital for the elucidating possible interactions between these different microbes, effects in these pathologies and possible new treatments. DESM provides an easy-to-use, and quick methodology to explore such networks of interactions between human anatomy, microbial networks and disease.

## CURRENT STATUS AND UPDATES

Up-to-date statistics of the DESM are available on the website. We intend to update DESM KBs on a six months basis. In the future we plan to extend our list of KBs and dictionaries and encourage users to provide feedback.

## AVAILABILITY AND REQUIREMENTS

KBs are accessible through the DESM portal (www.cbrc.kaust.edu.sa/desm) using any of the mainstream web browsers including Firefox, Chrome and Safari. As far as we know the only feature that has browser inter-compatibility issues is the network export option that is only available through Chrome. The use of DESM is free for academic and non-profit users.
